# 

**Published:** 1898-02

**Authors:** 


					﻿ESTABLISHED 1*55.	SUBSCRIPTION, *2.00.
ATLANTA
JWEDIGflh AMD SURGIGIUi
_________JOURNAL.______________
newseries. Atlanta, Ga., February, 1898. No. 12.
				

